# Anti-Melanogenesis and Photoprotective Effects of *Ecklonia maxima* Extract Containing Dieckol and Eckmaxol

**DOI:** 10.3390/md20090557

**Published:** 2022-08-30

**Authors:** Lei Wang, Jun-Geon Je, Hyun-Soo Kim, Kaiqiang Wang, Xiaoting Fu, Jiachao Xu, Xin Gao, You-Jin Jeon

**Affiliations:** 1 College of Food Science and Engineering, Ocean University of China, Qingdao 266003, China; 2 Department of Marine Life Sciences, Jeju National University, Jeju 63243, Korea; 3 National Marine Biodiversity Institute of Korea, Seocheon 33677, Korea; 4 Marine Science Institute, Jeju National University, Jeju 63333, Korea

**Keywords:** *Ecklonia maxima*, melanogenesis, UVB irradiation, photodamage

## Abstract

Seaweeds are potential ingredients in the cosmeceutical industry. Our previous study demonstrates that the phlorotannin-enriched extract of *Ecklonia maxima* (EME-EA) containing dieckol and eckmaxol possesses strong anti-inflammatory activity and suggests the cosmeceutical potential of EME-EA. In order to evaluate the cosmeceutical potential of EME-EA, the anti-melanogenesis and photoprotective effects of EME-EA were investigated in this study. EME-EA remarkably inhibited mushroom tyrosinase and melanogenesis in alpha-melanocyte-stimulating hormone-stimulated B16F10 cells. In addition, EME-EA significantly suppressed UVB-induced HaCaT cell death that was consistent with inhibition of apoptosis and reduction in scavenging intracellular reactive oxygen species. Furthermore, EME-EA significantly inhibited collagen degradation and matrix metalloproteinases expression in UVB-irradiated HDF cells in a concentration-dependent manner. These results indicate that EME-EA possesses strong anti-melanogenesis and photoprotective activities and suggest EME-EA is an ideal ingredient in the pharmaceutical and cosmeceutical industries.

## 1. Introduction

The skin plays an important role in the physiological functions of humans. Because the skin is the organ that is directly exposed to the environment, it experiences more stress than other organs, from environmental factors such as chemicals and ultraviolet (UV) irradiation [1]. These environmental factors cause skin damage such as oxidative damage, pigment synthesis disorder, and collagen degradation [2,3]. Humans are inescapably exposed to these environmental factors in their daily life. Thus, it is necessary to protect the skin against damage induced by environmental factors. In recent decades, the discovery of a natural ingredient and the development of it into a skincare product has attracted researchers’ attention. 

Seaweeds are rich in natural compounds such as phenolic compounds, polysaccharides, and proteins [4,5,6]. The phenolic compounds isolated from seaweeds have been reported to possess numerous health benefits such as antioxidant, immune-activating, UV protective, anti-obesity, and anti-inflammatory effects [7,8,9]. Therefore, seaweed extracts containing phenolic compounds were thought of as the potential ingredient in the functional-food, pharmaceutical, and cosmeceutical industries [10].

*Ecklonia maxima*, a brown seaweed, is typically found along the Southern Atlantic coast of Africa. *E. maxima* is one of the main raw seaweeds in the production of alginate worldwide. However, the bioactivities of natural compounds of *E. maxima* have not been investigated in depth. The phlorotannin-enriched extract of *E. maxima* (EME-EA) was prepared, and its anti-inflammatory effect was evaluated in our previous study [11]. The results indicated that EME-EA contains dieckol and eckmaxol and possesses strong anti-inflammatory activity [11]. These results suggested the cosmeceutical potential of EME-EA. However, the cosmeceutical effect of EME-EA has not been investigated so far. Thus, in the present study, we evaluate the anti-melanogenesis and UV protective effects of EME-EA. 

## 2. Results and Discussion

### 2.1. Anti-Melanogenesis Effect of EME-EA

Melanin is the main pigment that contributes to the color of the hair, eyes, and skin of humans. It plays an important role in the skin against photodamage. However, the overproduction of melanin can cause pigment disorders such as moles and freckles. Excessive melanogenesis is associated with the high activity of tyrosinase, and oxidative stress [12,13]. Tyrosinase is the key enzyme during melanin biosynthesis. Thus, an ingredient that can inhibit the activity of tyrosinase may possess the potential in anti-melanogenesis effect. 

**Figure 1:**
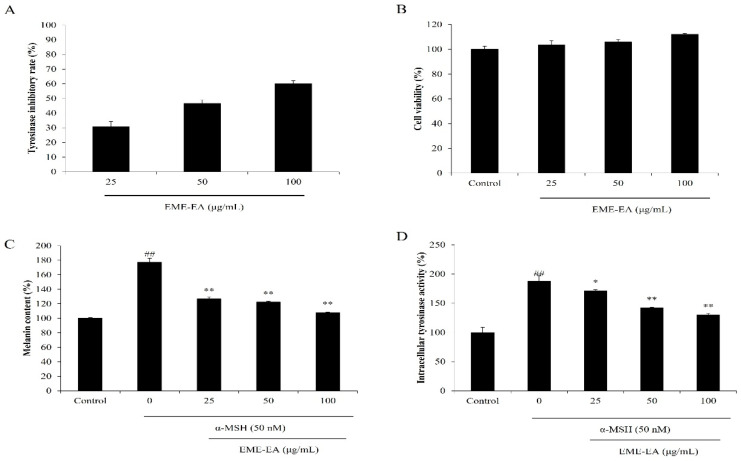
Anti-melanogenesis effect of EME-EA. (**A**) Tyrosinase inhibitory activity of EME-EA; (**B**) cytotoxicity of EME-EA on B16F10 cells; (**C**) melanin synthesis levels of B16F10 cells stimulated by α-MSH or co-treated with EME-EA; and (**D**) relative intracellular tyrosinase activities of B16F10 cells stimulated by α-MSH or co-treated with EME-EA. The data were expressed as the mean ± SE (*n* = 3). * *p* < 0.05, ** *p* < 0.01 as compared to α-MSH-treated group and ## *p* < 0.01 as compared to control group.

In the present study, the inhibitory effect of EME-EA on tyrosinase from mushroom was evaluated. As shown in Figure 1A, EME-EA inhibited 30.72, 46.58, and 60.04% of tyrosinase at the concentration of 25, 50, and 100 μg/mL, respectively. It demonstrated that EME-EA could inhibit tyrosinase in a concentration-dependent manner and suggested the potential of EME-EA on anti-melanogenesis effect. As shown in Figure 1B,C, EME-EA remarkably reduced the melanin content in alpha-melanocyte-stimulating hormone (α-MSH)-stimulated B16F10 cells without toxicity. Further results demonstrated that this effect was actioned by inhibiting the intracellular tyrosinase activity in α-MSH-stimulated B16F10 cells. These results indicated that EME-EA possesses the whitening effect and suggested the potential of EME-EA in the cosmeceutical industry.

### 2.2. Protective Effect of EME-EA against UVB-Induced Photodamage

Skin is directly exposed to environmental factors. UV irradiation was thought of as the primary factor causing skin damage. Overexposure to UV causes erythema, hyperpigmentation, wrinkling, and skin cancer [14,15]. According to the wavelength, UV can be classified into three subtypes that UVA (320~400), UVB (280~320 nm), and UVC (100~280 nm). Generally, all UVC and some UVB rays are absorbed by the Earth’s ozone layer. UVA and a small amount of UVB come to the surface of the Earth. Both UVA and UVB have long-term effects on humans. UVB is thought of as it produces more stress in humans than the other subtypes. UVB is characterized by its ability to penetrate the layers of the stratum corundum and epidermis, causing thickening of the epidermis, deep wrinkling, mottled discoloration, and a loss of elasticity. The above data displayed that EME-EA possesses a potent ROS scavenging effect and suggested its photoprotective potential. In order to further evaluate the cosmeceutical potential of EME-EA, the protective effect of EME-EA on UVB-induced photodamage in human epidermal keratinocytes (HaCaT cells) and human dermal fibroblasts (HDF cells) was investigated. 

**Figure 2:**
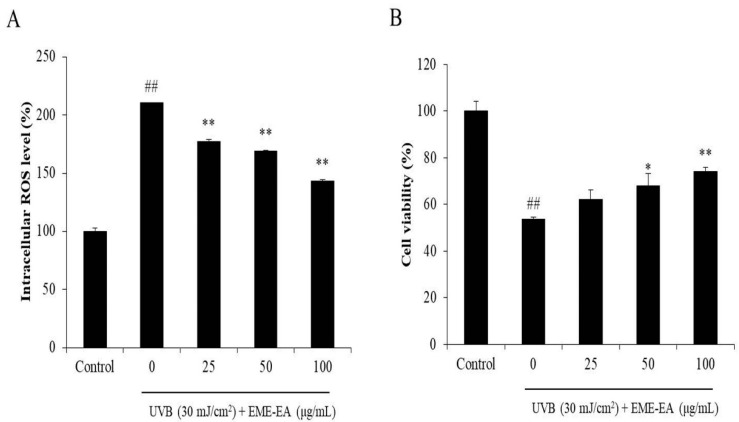
Protective effect of EME-EA against UVB-induced HaCaT cell damage. (**A**) The intracellular ROS levels of UVB-irradiated HaCaT cells; (**B**) the viability of UVB-irradiated HaCaT cells. The data were expressed as the mean ± SE (*n* = 3). * *p* < 0.05, ** *p* < 0.01 as compared to UVB-irradiated group and ## *p* < 0.01 as compared to control group.

**Figure 3:**
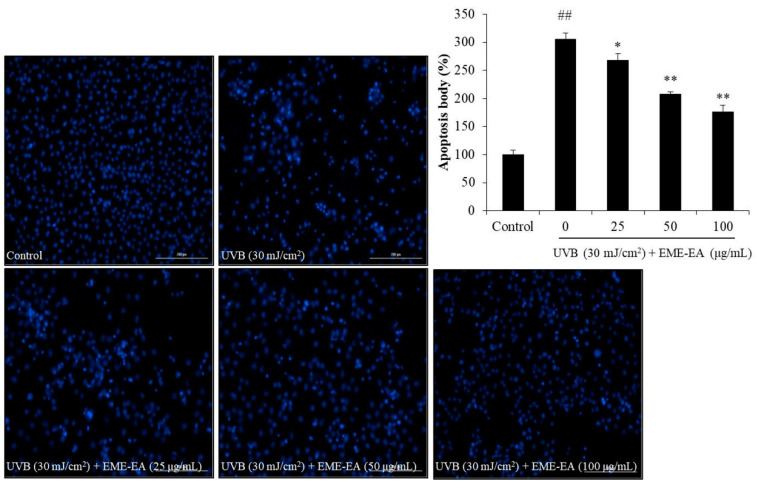
Protective effect of EME-EA against UVB-induced apoptosis in HaCaT cells. The relative levels of apoptosis were measured using Image J software. The data were expressed as the mean ± SE (*n* = 3). * *p* < 0.05, ** *p* < 0.01 as compared to UVB-irradiated group and ## *p* < 0.01 as compared to control group.

As Figure 2A shows, UVB irradiation increased the intracellular ROS level of HaCaT cells from 100% to 210.40%, whereas the intracellular ROS levels of UVB-irradiated HaCaT cells were decreased to 177.21, 169.22, and 143.08% by EME-EA at the concentration of 25, 50, and 100 μg/mL, respectively (Figure 2A). The viability of UVB-irradiated HaCaT cells was reduced to 53.51% compared to the non-irradiated cells (Figure 2B). However, EME-EA increased the viability of UVB-irradiated HaCaT cells to 62.20, 68.09, and 74.07% at concentrations of 25, 50, and 100 μg/mL, respectively (Figure 2B). Furthermore, UVB significantly stimulated apoptosis body formation in HaCaT cells (Figure 3). The apoptosis body formation of UVB-irradiated HaCaT cells was remarkably and concentration-dependently reduced by EME-EA (Figure 3). These results indicate that EME-EA significantly suppressed UVB-induced HaCaT cell death, consistent with the inhibition of apoptosis and a reduction in scavenging intracellular ROS. 

**Figure 4:**
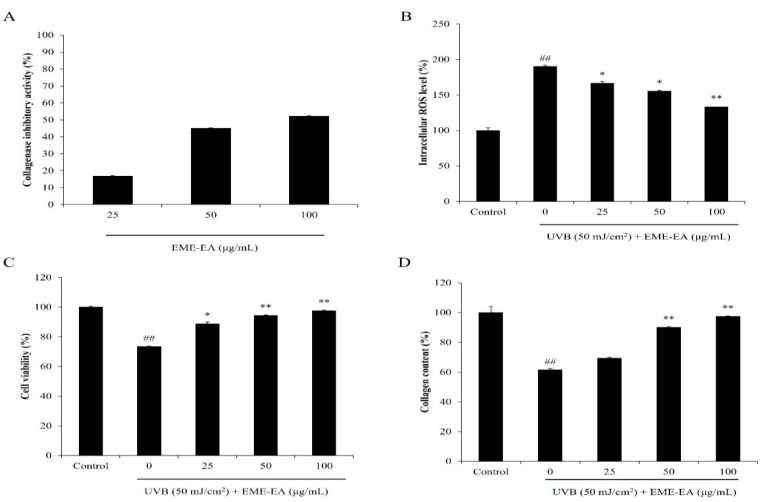
Protective effect of EME-EA against UVB-induced HDF cell damage. (**A**) Collagenase inhibitory effect of EME-EA; (**B**) the intracellular ROS levels of UVB-irradiated HDF cells; (**C**) the viability of UVB-irradiated HDF cells; and (**D**) collagen levels of UVB-irradiated HDF cells. The data were expressed as the mean ± SE (*n* = 3). * *p* < 0.05, ** *p* < 0.01 as compared to UVB-irradiated group and ## *p* < 0.01 as compared to control group.

**Figure 5:**
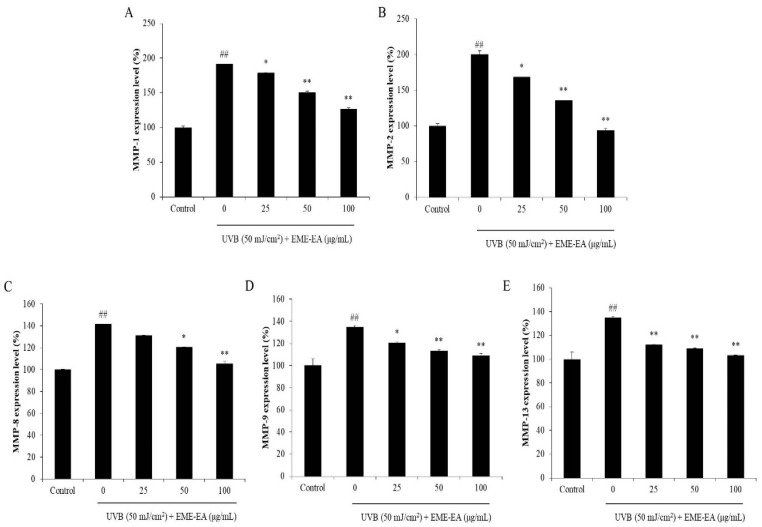
Inhibitory effect of EME-EA on MMPs expression in UVB-irradiated HDF cells. (**A**) MMP-1 expression level in UVB-irradiated HDF cells; (**B**) MMP-2 expression level in UVB-irradiated HDF cells; (**C**) MMP-8 expression level in UVB-irradiated HDF cells; (**D**) MMP-9 expression level in UVB-irradiated HDF cells; and (**E**) MMP-13 expression level in UVB-irradiated HDF cells. The data were expressed as the mean ± SE (*n* = 3). * *p* < 0.05, ** *p* < 0.01 as compared to UVB-irradiated group and ## *p* < 0.01 as compared to control group.

Collagenase is a protease that degrades collagen, which is the important structural and functional protein in the skin [16]. Degradation of collagen causes skin loss of structure and collapse of the dermal layer. Thus, a collagenase inhibitor may a potential candidate to suppress skin wrinkling. As shown in Figure 4A, EME-EA inhibited 16.82, 45.00, and 52.03% of collagenase at the concentration of 25, 50, and 100 μg/mL, respectively. This indicated that EME-EA possesses the inhibitory effect on collagenase, and it may a potential anti-wrinkle agent. Wherefore, the protective effect of EME-EA on UVB-stimulated dermic damage was investigated. As Figure 4B,C show, EME-EA not only decreased the intracellular ROS levels but also improved the viability of UVB-irradiated HDF cells. Both effects were concentration-dependent. As shown in Figure 4D, the collagen content of UVB-irradiated HDF cells was decreased to 61.57% compared to non-irradiated cells (100%). However, EME-EA increased the collagen content of UVB-irradiated HDF cells to 69.39, 90.07, and 97.23% at concentrations of 25, 50, and 100 μg/mL, respectively (Figure 4D). Furthermore, the matrix metalloproteinases (MMPs) levels of UVB-irradiated HDF cells were significantly increased, especially MMP-1 and MMP-2 (Figure 5). However, the MMPs levels of UVB-irradiated HDF cells were remarkably reduced by EME-EA treatment in a concentration-dependent manner (Figure 5). These results suggest that EME-EA has the potential to inhibit skin wrinkling, displayed in suppressing oxidative damage, preventing collagen degradation, and inhibiting MMPs expression in HDF cells, and further indicated the cosmeceutical potential of EME-EA.

In summary, the present study demonstrates that EME-EA possesses anti-melanogenesis and photoprotective effects and suggested its potential to make a skincare product in the cosmeceutical industry.

## 3. Materials and Methods

### 3.1. Chemicals and Reagents

Ham's nutrient mixtures medium (F-12 medium), trypsin-EDTA, Dulbecco's modified Eagle medium (DMEM), penicillin-streptomycin (P/S), Roswell Park Memorial Institute-1640 (RPMI-1640) medium, and fetal bovine serum (FBS) were purchased from Gibco-BRL (Grand Island, NY, USA). The MTT, DCFH2-DA, α-MSH, and the ELISA kits were purchased from Sigma (St. Louis, MO, USA). All other chemicals used in this study were of analytical grade.

### 3.2. Preparation of EMEEA

The EME-EA was prepared in our previous study [11]. In brief, the lyophilized *E. maxima* powder was extracted by 80% MeOH. The crude extract fractioned by n-hexane, chloroform, and ethyl acetate. The ethyl acetate fraction (EME-EA) was obtained. The main compounds of EME-EA were characterized as dieckol and eckmaxol [11].

### 3.3. Evaluation of Enzyme Inhibitory Effect of EME-EA

The inhibitory effect of EME-EA on tyrosinase was measured according to the protocol described in the previous study [17]. In addition, the inhibitory effect of EME-EA on collagenase was measured according to the methods described by Wang et al. [16].

### 3.4. Maintenance of Cell Lines

B16F10 cells were cultured in DMEM (10% of FBS and 1% of P/S) and seeded at a density of 1 × 10^5^ or 3 × 10^4^ cells/mL for experiments. HaCaT cells were cultured in DMEM (10% of FBS and 1% of P/S) and seeded at a density of 1 × 10^5^ cells/mL for experiments. HDF cells were cultured in the medium mixed with F-12 and DMEM (1:3) (10% of FBS and 1% of P/S) and seeded at a concentration of 5.0 × 10^4^ cells/mL for experiments. 

### 3.5. Determination of the Effect of EME-EA on α-MSH-Stimulated Melanogenesis

B16F10 cells were seeded in a six-well plate and incubated for 24 h. Cells were treated with 25, 50, and 100 μg/mL EME-EA and stimulated with 50 nM α-MSH. The α-MSH-stimulated cells were harvested after 72 h incubation. Then, the melanin content and intracellular tyrosinase activity of α-MSH-stimulated cells were measured according to the method described by Heo. et al. [18].

### 3.6. Determination of the Effect of EME-EA on Photodamage Induced by UVB Irradiation in HaCaT and HDF Cells

HaCaT cells were seeded and treated with EME-EA. The photoprotective effect of EME-EA in HaCaT cells was determined by evaluating the intracellular ROS, cell viability, and apoptosis according to the methods described in the previous study [19]. HDF cells were seeded and treated with EME-EA. EME-EA-treated cells were exposed to UVB (50 mJ/cm^2^). The intracellular ROS level and the viability of UVB-irradiated HDF cells were measured by DCF-DA assay and MTT assay, respectively [16]. The collagen level and the MMPs expression levels were assessed by ELISA [20].

### 3.7. Statistical Analysis

The experiments were performed in triplicate, and the data are expressed as the mean ± standard error (SE). One-way ANOVA was used to compare the mean values of each treatment in SPSS 20.0. Significant differences between the means were identified by the Turkey test. 

## 4. Conclusions

In the present study, the cosmeceutical effects of the *E. maxima* extract (EME-EA) were investigated. The results suggested that EME-EA possesses the cosmeceutical potential displayed in inhibiting melanogenesis and suppressing photodamage. The present study suggests that EME-EA could be used as a cosmetic agent to prevent skin aging.

## Data Availability

Not applicable.
